# Prevalence of Lassa Virus Disease (LVD) in Nigerian children with fever or fever and convulsions in an endemic area

**DOI:** 10.1371/journal.pntd.0005711

**Published:** 2017-07-03

**Authors:** Odigie C. Akhuemokhan, Rosemary O. Ewah-Odiase, Nosa Akpede, Jacqueline Ehimuan, Donatus I. Adomeh, Ikpomwonsa Odia, Sylvia C. Olomu, Meike Pahlmann, Beate Becker-Ziaja, Christian T. Happi, Danny A. Asogun, Sylvanus A. Okogbenin, Peter O. Okokhere, Osagie S. Dawodu, Irekpono U. Omoike, Pardis C. Sabeti, Stephan Günther, George O. Akpede

**Affiliations:** 1 Department of Paediatrics, Irrua Specialist Teaching Hospital, Irrua, Edo State, Nigeria; 2 Institute of Lassa Fever Research and Control, Irrua Specialist Teaching Hospital, Irrua, Edo State, Nigeria; 3 Bernhard-Nocht-Institute for Tropical Medicine, WHO Collaborating Centre for Arboviruses and Hemorrhagic Fever Reference and Research, Department of Virology, Bernhard-Nocht-Str. 74, Hamburg, Germany; 4 Malaria Research Laboratory, College of Medicine, University of Ibadan, Ibadan, Oyo State, Nigeria; 5 Department of Obstetrics and Gynaecology, Irrua Specialist Teaching Hospital, Irrua, Edo State, Nigeria; 6 Department of Medicine, Irrua Specialist Teaching Hospital, Irrua, Edo State, Nigeria; 7 Department of Organismic Biology, Broad Institute, Harvard University, Cambridge, Massachusetts, United States of America; 8 Department of Paediatrics, Faculty of Clinical Sciences, College of Medicine, Ambrose Alli University, Ekpoma, Edo State, Nigeria; School of Veterinary Medicine University of California Davis, UNITED STATES

## Abstract

**Background:**

Convulsions with fever in children are a common neurologic emergency in the tropics, and determining the contribution of endemic viral infections can be challenging. In particular, there is a dearth of data on the prevalence and clinical differentiation of Lassa virus disease (LVD) in febrile children in endemic areas of Nigeria, which has multiple lineages of the virus. The aim of this study was to determine the prevalence and presentation of LVD in febrile children with and without convulsions.

**Methodology/Principal findings:**

This was a prospective study of consecutive febrile children aged ≥1 month– 15 years admitted to the Children’s Emergency Room of Irrua Specialist Teaching Hospital over a period of 1 year. Febrile children with convulsions (Cases) were compared with those without convulsions (Controls). LVD was defined by the presence of a positive Lassa virus RT-PCR test. Rates were compared between groups using χ2 or Fisher’s exact tests and *p* <0.05 taken as significant. 373 (40.9%) of 913 admissions had fever. Of these, 108/373 (29%) presented with convulsions. The overall prevalence of LVD was 13/373 (3.5%; 95% CI = 1.9%, 5.7%) in febrile admissions, 3/108 (2.8%) in Cases and 10/265 (3.8%) in Controls [(Odds Ratio (95% Confidence Interval) (OR (95% CI)) of LVD in Cases versus Controls = 0.73 (0.2, 2.7)]. Only vomiting (OR (95% CI) = 0.09 (0.01, 0.70)) and bleeding (OR (95% CI) = 39.56 (8.52, 183.7)) were significantly associated with an increased prevalence of LVD.

**Conclusions/Significance:**

LVD is an important cause of fever, including undifferentiated fever in children in endemic areas, but it is not significantly associated with convulsions associated with fever. Its prevalence, and lack of clinical differentiation on presentation, underscores the importance of a high index of suspicion in diagnosis. Screening of febrile children with undifferentiated fever in endemic areas for LVD could be an important medical and public health control measure.

## Author summary

There has, perhaps, been undue focus on malaria as a cause of childhood fever and convulsions, often with delayed/missed diagnosis of other serious prevalent infections, and correspondingly very little published data on the contribution of Lassa virus disease (LVD) in endemic areas. There is also very little published data on the contribution of LVD to childhood morbidity and mortality in Nigeria, a large LVD-endemic country that has in circulation 3 of the 4 currently known lineages of the Lassa virus. This study was carried out to address these gaps. The results should also be of relevance in the formulation of policies for the treatment and control of viral haemorrhagic fevers. The prevalence of LVD was 5.4% among children with clinically undifferentiated fever (n = 243); 3.9% among those with convulsions associated with fever (n = 77) and 6.0% among those with fever but no convulsions (n = 166). The results underscore the importance of LVD as a cause of acute undifferentiated fever. The results also underscore the need of diagnostic testing for LVD in children with acute undifferentiated fever in endemic areas in order to facilitate control, including the prevention of nosocomial transmission.

## Introduction

The causes of convulsions associated with fever (CAWF), a common paediatric emergency worldwide [1,2], are dependent on age, climate and clinical presentation [3]. Viral infections in temperate countries [4,5], and malaria in tropical countries [3,6–8] are the main aetiologic agents in febrile convulsions but bacterial infections are nonetheless also important causes in both temperate and tropical countries [9–13]. However, while the prevalence, presentation and outcome of malaria and bacterial infections as causes of CAWF in the tropics have been fairly well defined [3,6–8,11], there are only a few reports on the contribution of viral infections [14,15]. Specifically, there is little information on the contribution of Lassa virus disease (LVD).

More broadly, reports of the prevalence of LVD in children are few and knowledge of the clinical manifestations limited. In addition, a majority of the studies on the prevalence and clinical features of LVD in children and adults are from the West African countries of Sierra Leone, Liberia and Guinea [16–20], as are the few reports on paediatric LVD [21–23], and little is known about the situation in Nigeria. There are 4 major lineages reported thus far from sequencing of the Lassa virus (LASV): 3 are in circulation in Nigeria and 1 in other West African countries [24,25]. Whether the lineages are associated with different clinical manifestations is largely unknown.

Nigeria is one of the LVD-endemic countries in West Africa and reports indicate that it may account for up to 6% of febrile admissions in endemic areas in the country [26]. Its initial clinical manifestations are difficult to differentiate from those of other common febrile illnesses, such as malaria [16,18,27], and a high index of clinical suspicion is required in the diagnosis [27]. Convulsions have been described in LVD as a possible marker of disease severity and a frequent herald of death alongside coma [28,29]. These convulsions could be caused by several mechanisms, including infection of neural tissue, fever and metabolic changes among others as in other viral haemorrhagic fevers [30,31].

Few reports, if any, are available from endemic areas on the contribution of LVD to the common neurologic emergency of CAWF in children, and its true prevalence in childhood CAWF has not been determined. Addressing this should add to our understanding of the manifestations of this emerging/remerging infectious disease with respect to CAWF in the tropics. Perhaps, undiagnosed LVD could contribute to the relatively poor outcome of febrile convulsions in endemic areas in the tropics.

Several factors help account for why so little is known about the role of LVD in childhood CAWF: the low awareness of LVD as a possible aetiology in childhood fever and CAWF; the difficulties in differentiating it from other common causes of fever; and the lack of accessible diagnostic facilities. However, better understanding of the role of LVD could provide a useful guide to clinicians in the management of this common neurologic emergency. This is aside its public health importance. This study therefore sought to address these gaps in knowledge. Specifically, we had two aims: to systematically determine the prevalence of LVD in children with CAWF compared to those with fever but no convulsions, and to compare the clinical manifestations of LVD with that of other common febrile illnesses in children. In addition, we sought to determine the current status of malaria as a contributor to CAWF in endemic areas.

## Methods

### Ethics statement

This study was approved by the Research and Ethics Committee of Irrua Specialist Teaching Hospital, via approval number ISTHREC09/14/12/2009 dated 7^th^ January 2010.

### Patients and methods

This was a descriptive, prospective case-control study of consecutive febrile children with convulsions presenting to the Children Emergency Room (CHER), Irrua Specialist Teaching Hospital (ISTH), Irrua, Edo State, NIgreia. Irrua is the headquarters of Esan-Central Local Government Area (LGA), Edo State, one of the local government areas with the highest prevalence of LVD in Nigeria [32]. The Research and Ethics Committee of ISTH approved the study, and written informed consent was obtained from the parents/guardians of the children involved in the study. The institutional review boards of the collaborating institutions from which the co-authors were drawn also approved the study.

Enrolment consisted of all consecutive admissions of children aged >1 month to ≤15 years, admitted over a period of one year (December 1^st^, 2009 to November 30^th^, 2010) into the CHER with complaints of fever or fever and convulsions. The attending physician had responsibility for the admissions, which were not associated with any particular diagnosis, including LVD, and the authors had no input in the selection of patients for admission.

The inclusion criteria for the study included temperature ≥38°C and written informed consent given by the parents or guardians. On admission a detailed history, clinical examination and laboratory tests were undertaken for each patient, after which the data obtained were entered into a standardized form. For the purpose of this study, a ‘Case’ was defined as a child aged ≥1month to ≤15 years, with temperature ≥38°C on admission or a history of fever + temperature ≥38°C within 8 hours of admission, and a history of convulsions during the illness. A ‘Control’ met the same criteria but had no history of convulsions. Both Cases and Controls were given the standard management appropriate for their diagnosis. Patients with LVD were given intravenous ribavirin at 33mg/kg body weight stat, and maintained on 16mg/kg body weight/dose 6 hourly for the first 4 days and thereafter 8mg/kg body weight/dose 8 hourly for another 6 days. In all, therapy with ribavirin was for 10 days [33]. Patients with a diagnosis of sepsis were commenced on empiric antibiotics after appropriate samples had been taken for culture. Thus patients with a diagnosis of meningitis were commenced on intravenous ceftriaxone and those with severe sepsis on ceftriaxone, or cefotaxime, or ceftazidime, and an aminoglycoside (gentamicin or amikacin), pending the receipt of culture results.

The history included presenting complaints, drug history, history of recent immunizations, and history of the pattern, frequency and duration of convulsions, if any. Physical examination included a careful examination of the skin and mucous membranes for petechiae, purpura or ecchymosis and examination to exclude a focus of infection such as pharyngotonsilitis, otitis media, pneumonia and meningitis. Presence of signs of meningeal irritation (nuchal rigidity, Kerning’s and Brudzinski’s signs and/or bulging and tense fontanel) was considered as indicative of meningitis. The level of consciousness was assessed using the Blantyre [34] and Glasgow [35] Coma Scales, depending on the child’s age.

Blood, cerebrospinal fluid (CSF) and urine samples were collected on admission and processed at the respective service laboratories of the hospital using standard methods. The laboratory tests done included LASV-reverse transcriptase-polymerase chain reaction (RT-PCR) test, haematocrit, white blood cell count (WBC), dipstick urinalysis, microscopic urinalysis [36], blood film for malaria parasites (MP), random blood sugar, blood urea nitrogen, serum creatinine and blood culture. Lumbar puncture and CSF examination were carried out on all children who presented with fever and convulsions [37–39] as well as those with signs of meningeal irritation, except where there were contraindications to lumbar puncture [40,41]. Blood culture was carried out by inoculation of blood drawn aseptically from a peripheral vein into a pair of culture bottles containing glucose broth and sodium thioglycollate, which were then incubated at 37°C. Routine Gram staining and subcultures onto MacConkey and blood agar plates were carried out at 2 and 7 days or whenever a growth became evident. CSF samples were collected in sterile bottles and cultured by inoculation onto blood, chocolate and MacConkey agar plates, and incubated at 37°C for 48 hours under both aerobic and anaerobic conditions. Both blood and CSF cultures were processed manually as our hospital does not, so far, have automated systems.

Malaria was defined as the presence of asexual forms of MP in a peripheral blood smear. Bacterial meningitis was defined as the presence of elevated CSF WBC count (>5/mm^3^) plus CSF/blood glucose ratio <40% or CSF glucose <40 mg/dl and CSF protein >40 mg/dl plus a positive CSF Gram stain or culture [37,42,43]. Possible bacterial meningitis was defined as the presence of elevated CSF WBC count (>5/mm^3^) plus CSF/blood glucose ratio <40% or CSF glucose <40 mg/dl and CSF protein >40 mg/dl but negative CSF Gram stain or culture [43]. The use of CSF white blood cell count of >5/mm^3^ as the threshold for the diagnosis of meningitis could be attended with increased sensitivity in very young infants in whom the normal counts are higher. However, the diagnosis of possible bacterial meningitis was not based only on this parameter but on both total and differential white blood cell count and the presence of typical biochemical changes [42,44,45], which are known to have a high sensitivity and specificity for bacterial meningitis [44,45]. We adopted the term ‘possible bacterial meningitis’ in deference to the lack of confirmatory findings by way of positive Gram stain or culture or antigen tests [43].

A child was taken to have unidentified infection in the absence of a focal infection, as indicated by the presence of localising signs, malaria parasitaemia, positive blood culture, bacterial or possible bacterial meningitis or a positive LASV-RT-PCR test, or clinical explanation/s for the febrile illness such as gastroenteritis, HIV/AIDS, or upper respiratory tract infection. Severe anaemia was defined as haematocrit ≤20% [46], leucopaenia as peripheral WBC <3000/mm^3^, leukocytosis as WBC ≥15000/mm^3^ [9] and hypoglycaemia as blood glucose ≤2.2 mmol/L [47].

All the febrile admissions had LASV-RT-PCR test, which was carried out at the Research and Diagnostic Laboratory of the Institute of Lassa Fever Research and Control (ILFRC), ISTH. The ribonucleic acid (RNA) was prepared from 140 μL of serum and conventional PCR tests carried out as described elsewhere [48]. LASV-RT-PCR test was carried out on the serum of both Cases and Controls. RNA was purified with QIA amp viral RNA KIT (Qiagen, Hilden, Germany). RT-PCR was performed with superscript II RT/Platinum taq polymerase 1–step reagents (Invitrogen, Karlsruhe, Germany). This afforded a 95% detection limit of 2,500 copies/ml, which is highly sensitive. A positive LASV-RT-PCR test was taken as confirmatory of the diagnosis of LVD [48].

### Data analyses

Children with fever were classified into Cases and Controls. Cases were sub-classified into two groups: children with febrile convulsions (FC) [1] and children with non-febrile convulsions (NFC). The latter included children outside the defined age range of 6 months– 5 years for FC [1] and children with convulsions associated with central nervous system infection as indicated by abnormal findings on CSF examination. Both Cases and Controls were further classified based on clinical presentation into 2 groups: clinically differentiated fever (children with fever with localizing signs of infection and children without localizing signs but with plausible clinical explanation/s for the fever, such as the development of fever within 24–48 hours of vaccination with diphtheria, pertussis and tetanus (DPT) vaccine or a clinical diagnosis of pertussis) and children with fever without localizing signs (undifferentiated fever).The statistical significance of differences in the prevalence of LVD between the groups, and significance of the association of symptoms and signs with LVD, was determined using χ2 test with Yates’ correction or Fisher Exact Test as appropriate. Two-tailed *p* values <0.05 were taken as significant.

## Results

Nine hundred and thirteen children were admitted to the CHER during the study. Three hundred and seventy-three (40.9%) had fever. Of these, 108 (29%) were Cases and 265 (71%) Controls. Sixty-one (56.5%) Cases had FC and 47 (43.5%) NFC. Seventy-six (70.4%) had generalized, 13 (12.0%) focal and 19 (17.6%) unclassifiable convulsions. The generalized convulsions were tonic-clonic in 64 (59.3%), tonic in 11 (10.2%), and clonic in 1 (0.9%). Four (3.7%) children had status epilepticus.

The differences between the general characteristics of Cases and Controls in terms of seasonality, age, gender, and general clinical and laboratory profile are shown in Table 1. More Cases presented during the rainy season months of May–October (60% versus 32%, *p* <<0.001), had severe anaemia (23% versus 14%, *p* = 0.045) and proteinuria (12% versus 8%, *p* = 0.004) and had lumbar puncture on admission (97% versus 49%, *p* <<0.001). The other differences between the Cases and Controls were not statistically significant.

**Table 1:** Seasonality, age, gender and general clinical and laboratory profile of Cases and Controls.

Feature	No. (%) of children (n = 373)	No. (%) Cases (n = 108)	No. (%) Controls (n = 265)	OR (95% CI)	*p*
**Presented during rainy season**	151 (40.5)	65 (60.2)	86 (32.4)	3.15 (1.98, 5.00)	<<0.001
**Age ≤5 years**	286 (76.7)	86 (79.6)	200 (75.5)	1.27 (0.74, 2.19)	0.468
**Gender = Male**	218 (58.5)	65 (60.2)	153 (57.7)	1.11 (0.70, 1.75)	0.749
**Undifferentiated fever**	243 (65.2)	77 (71.3)	166 (62.6)	1.48 (0.91, 2.41)	0.141
**LP done on admission** **CSF abnormalities present**	235 (63.0) 17 (4.6)	105(97.2)* 7/105 (6.7)	130 (49.1) 10 (3.8)	36.35 (11.25, 117.4) 1.82 (0.67, 4.92)	<<0.001 0.353
**Severe anaemia** **Hct ≤20%** **Hct ≤15%**	62 (16.6) 51 (13.7)	25 (23.2) 14 (13.0)	37 (14.0) 37 (14.0)	1.86 (1.05, 3.27) 0.92 (0.47, 1.78)	0.045 0.92
**Leukopaenia**	10 (2.7)	2 (1.9)	8 (3.0)	0.61 (0.13, 2.90)	0.818
**Leukocytosis**	102 (27.3)	29 (26.9)	73 (27.6)	0.97 (0.58, 1.60)	0.993
**Hypoglycaemia**	10 (2.7)	2 (1.9)	8 (3.0)	0.61 (0.13, 2.90)	0.818
**Proteinuria**	20 (5.4)	12 (11.1)	8 (3.0)	4.02 (1.59, 10.12)	0.004
**Haematuriay**	11 (3.0)	5 (4.6)	6 (2.3)	2.10 (0.63, 7.02)	0.369
**Positive EMUA**	2 (0.5)	0	2 (0.8)	NA	>0.999

OR (95% CI) = Odds Ratio (95% Confidence Interval) of feature in Cases versus Controls; LP = lumbar puncture; CSF = cerebrospinal fluid; Hct = haematocrit; EMUA = enhanced microscopic urinalysis; NA = not applicable.

*Three children presented with status epilepticus and could not undergo LP on presentation but had florid signs of meningeal irritation. These children were excluded from this analysis, hence N <108.

### Pattern of fever in febrile children and pattern of illnesses in children with differentiated fever

Overall, 130 (34.9%) children had clinically differentiated fever, and 243 (65.1%) undifferentiated fever. The proportion of children with differentiated fever was lower among the Cases (28.7%) than among the Controls (37.4%) but the difference did not attain statistical significance (*p* = 0.141).

The pattern of illnesses in children with differentiated fever is shown in Table 2. Overall, 52.3% had focal extra-cranial infections, and 15.4% possible bacterial meningitis while 32.3% had neither infection but had other clinical explanations for their febrile illnesses. None had confirmed bacterial meningitis. The focal extra-cranial infections included bronchopneumonia, acute otitis media, acute suppurative otitis media, and abscesses. The diagnosis in children with neither focal extra-cranial infections nor meningitis but with clinical explanation/s for their febrile illnesses included gastroenteritis, HIV/AIDS, upper respiratory tract infection, myocarditis, organophosphorus insecticide poisoning, diabetic ketoacidosis, lymphoma, pertussis, cholestatic jaundice, chronic renal failure, Kawasaki disease, severe malnutrition, neuroblastoma, and pelvic inflammatory disease. The proportion of children with possible bacterial meningitis was significantly higher among the Cases (32.3% versus 10.1%, *p =* 0.01) (Table 2). The difference between the proportions of Cases and Controls with focal extra-cranial infections and the other illnesses was not statistically significant (Table 2). Children with possible bacterial meningitis had a significantly higher mean total CSF white blood cell count (*p* = 0.01), CSF glucose (*p* <0.001), CSF/simultaneous blood glucose ratio (*p* <<0.001) and CSF protein (*p* <0.001) (Table 3).

**Table 2:** Pattern of illnesses in febrile children with differentiated fever.

Illness	No. (%) of children	No. (%) Cases	No. (%) Controls	OR (95% CI)	*p*
**Focal extra-cranial infections**	**68 (52.3)**	**12 (38.7)**	**56 (56.6)**	**0.49 (0.21, 1.11)**	**0.126**
**Possible bacterial meningitis**	**20 (15.4)**	**10 (32.3)**	**10 (10.1)**	**4.24 (1.56, 11.49)**	**0.010**
**Miscellaneous**	42 (32.3)	**9 (29.0)**	**33 (33.3)**	**0.82 (0.34, 1.97)**	**0.821**
**Total**	**130 (100)**	**31 (100.0)**	**99 (100.0)**	**NA**	**NA**

OR (95% CI) = Odds Ratio (95% Confidence Interval) of feature in Cases versus Controls; NA = not applicable

χ2 = 9.11, degrees of freedom = 2, *p* = 0.011

**Table 3:** Mean CSF white blood cell count, glucose, CSF/blood glucose ratio and CSF protein in children with versus those without meningitis.

CSF parameter	Mean (SD)/95% CI in children with possible bacterial meningitis (n = 20)	Mean (SD)/95% CI in children without meningitis (n = 353)	*p*
**Total WBC/mm^3^**	330.5 (45.8)/60.1, 600.9	5.9 (6.2)/5.0, 6.8	0.01
**CSF glucose, mg/dl**	36.9 (11.0)/30.4, 43.4	51.4 (15.7)/49.2, 53.6	<0.001
**CSF/blood glucose ratio, %**	28.1 (0.1)/28.0, 28.2	75.3 (11.2)/73.7, 76.9	<<0.001
**CSF protein, mg/dl**	94.5 (17.6)/84.1, 104.8	20.1 (67.1)/10.7, 29.5	<0.001

CSF = cerebrospinal fluid; 95% CI = 95% Confidence Interval

### Prevalence of Lassa virus disease and malaria parasitaemia in children with undifferentiated fever

The overall contribution of LVD and malaria parasitaemia in children with undifferentiated fever is shown in Table 4. The overall prevalence of LVD in undifferentiated fever was 5.4% (95% CI = 3.0%, 8.7%) while the prevalence of malaria parasitaemia was 35.8% (95% CI = 29.96%, 41.98%). Four children (1.6%), including 4/13 (30.8%) with LVD or 4/87 (4.6%) with malaria had LVD/malaria comorbidity. All the malaria parasites were *Plasmodium falciparum*. The youngest child with LVD was 6 months old and the oldest 7 years.

**Table 4:** Pattern of infections in children with undifferentiated fever.

**Infection**	**No. (%) of children**	**No. (%) Cases**	**No. (%) Controls**	**OR (95% CI)**	***p***
**LVD**	9 (3.7)	0	9 (5.4)		
**LVD + MP**	4 (1.6)	3 (3.9)	1 (0.6)		
**Total LVD**	13 (5.4)	3 (3.9)	10 (6.0)	0.63 (0.17, 2.37)	0.729
**Malaria parasitaemia**	83 (34.2)	47 (61.0)	36 (21.7)	5.66 (3.14, 10.19)	<<0.001
**Unidentified**	147 (60.5)	27 (35.1)	120 (72.3)	0.21 (0.12, 0.37)	<<0.001
**Total**	243 (100.0)	77 (100.0)	166 (100.0)	NA	NA

OR (95% CI) = Odds Ratio (95% Confidence Interval) of feature in Cases versus Controls; LVD = Lassa virus disease; MP = malaria parasitaemia; NA = not applicable

χ2 = 36.34, degrees of freedom = 2, *p* <<0.001

There was no significant difference between the overall prevalence of LVD in Cases and Controls (3.9% versus 6.0%, *p* = 0.729). However, the prevalence of LVD/malaria parasitaemia was significantly higher among the Cases than the Controls with LVD (3.9% versus 0.6%, *p* = 0.028). Two of the 3 Cases with LVD had generalised tonic-clonic and 1 focal convulsions; the numbers were too small for further analysis. The prevalence of malaria parasitaemia was significantly higher among the Cases (61.0% versus 21.7%, *p* <<0.001) while the prevalence of children with unidentified infections was significantly lower among the Cases (35.1% versus 72.3%, *p* <<0.001).

### Presentation and pattern of illnesses in Cases with febrile versus non-febrile convulsions

Sixty-one Cases (56.5%) had FC and 47 (43.5%) NFC, giving a ratio of FC: NFC of 1.3: 1. The prevalence of undifferentiated fever and pattern of infections in children with FC versus NFC is shown in Table 5. By definition, none of the children with FC had meningitis whereas 10 (21.3%) of those with NFC had possible bacterial meningitis. Children with FC had a significantly higher prevalence of undifferentiated fever (80.3% versus 59.6%, *p* = 0.032) but there was no significant difference between the two groups of children in the pattern of infections among children with undifferentiated fever.

**Table 5:** Prevalence of undifferentiated fever and contribution of LVD and malaria parasitaemia to undifferentiated fever in Cases with febrile versus non-febrile convulsions.

Feature	All Cases (n = 108)	Cases with FC (n = 61)	Cases with NFC (n = 47)	OR (95% CI)	*p*
**No. (%) with UF**	77 (71.3)	49 (80.3)	28 (59.6)	2.77 (1.17, 6.54)	0.032
**No. (%) with differentiated fever**	31 (28.7)	12 (19.6)	19 (40.4)		
**Pattern of infections in UF**					
***No*. *(%) with LVD ± MP***	3/77 (3.9)	1/61 (1.6)	2/47 (4.3)	0.38 (0.03, 4.27)	0.818
***No*. *(%) with MP***	47/77 (61.0)	31/61 (50.8)	16/47 (34.0)	2.00 (0.91, 4.39)	0.122
***No*. *(%) with UI***	27/77 (35.1)	17/61 (27.9)	10/47 (21.3)	1.43 (0.58, 3.50)	0.575

FC = febrile convulsions, NFC = non-febrile convulsions; OR (95% CI) = Odds Ratio (95% Confidence Interval) of feature in febrile versus non-febrile convulsions; LVD = Lassa virus disease; MP = malaria parasitaemia; UF = undifferentiated fever; UI = unidentified infections.

### Association between the prevalence of LVD and bio data, season, and non-localising presenting signs and symptoms

Cases and Controls were merged in analysis for the purpose of determining the association between age, gender, season and clinical presentation and the prevalence of LVD because the number of Cases (n = 3) as well as the number of Controls (n = 10) with LVD was small. The association between the prevalence of LVD and age, gender, season and clinical presentation in terms of the duration of fever on presentation, clinical status with regard to the presence versus absence of localising signs of infection, initial diagnosis and differential diagnosis and result of urinalysis is shown in Table 6. Overall, 9.4% febrile admissions presented with a history of fever ≥7 days, 76.4% were reported as acutely ill looking on presentation, and 77.5% had no localizing signs of infection, while Lassa fever was the initial diagnosis or differential diagnosis on admission in 2.9%. The association between the prevalence of LVD and the initial diagnosis/differential diagnosis was highly significant (*p* <<0.001) whereas none of the other associations attained statistical significance (Table 6).

**Table 6:** Association between the prevalence of LVD and age, gender, season, and clinical status on presentation.

**Status on presentation**	**No. (%) of febrile admissions (N = 373)**	**No. (%) with LVD (n = 13)**	**No. (%) with other febrile illnesses (n = 360)**	**OR (95% CI) of LVD in children with versus without stated status**	*p*
**Age ≤5 years**	286 (76.7)	10 (76.9)	276 (76.7)	1.01 (0.27, 3.77)	>0.999
**Gender = Male**	215 (57.6)	9 (69.2)	206 (57.2)	1.68 (0.51, 5.56)	0.565
**Presented in rainy season**	195 (52.3)	7 (53.8)	188 (52.3)	1.07 (0.35, 3.24)	0.867
**History of convulsions**	108 (29.0)	3 (23.1)	105 (29.2)	0.73 (0.20, 2.7)	0.903
**Generalized convulsions**	64/108 (59.3)	2/3 (66.7)	62/105 (59.1)	1.39 (0.12, 15.78)	>0.999
**Fever ≥7 days on presentation**	35 (9.4)	1 (7.7)	34 (9.4)	0.80 (0.10, 6.33)	,>0.999
**Acutely ill looking**	285 (76.4)	13 (100.0)	272 (75.6)	NA	0.057
**No localising signs of infection**	289 (77.5)	13 (100.0)	276 (76.7)	NA	0.068
**Initial diagnosis or differential diagnosis of Lassa fever**	11 (2.9)	9 (69.2)	2 (0.6)	N/A	<<0.001
**Proteinuria present**	20 (5.4)	2 (15.4)	18 (5.0)	3.46 (0.71, 16.76)	0.299
**Haematuria present**	11 (2.9)	2 (15.4)	9 (2.5)	7.09 (1.37, 36.75)	0.103

OR (95 CI) = Odds Ratio (95% Confidence Interval); NA = not applicable; N/A = not appropriate

The association between non-localizing symptoms and signs on presentation and the prevalence of LVD is shown in Table 7. Only the lack of a history of vomiting (*p* = 0.01) and presence of bleeding manifestations (*p* <0.001) had a statistically significant association with LVD (Table 7).

**Table 7:** Presenting features in children with LVD versus children with other infections.

**Presenting signs and symptoms**	**No. (%) in children with**			
	LVD (N = 13)	Other febrile illnesses (N = 360)	OR (95% CI)	*p*
**Sore throat**	0	4 (1.1)	NA	>0.999
**Muscle ache**	0	15 (4.2)	NA	>0.999
**Headache**	0	45 (12.5)	NA	0.365
**Chest pain**	0	10 (2.8)	NA	>0.999
**Abdominal pain**	1 (7.7)	51 (14.2)	0.51 (0.06, 3.97)	0.874
**Poor appetite**	1 (7.7)	73 (20.3)	0.33 (0.04, 2.58)	0.466
**Yellowness of the eyes**	0	26 (7.2)	NA	0.769
**Nausea**	0	23 (6.4)	NA	0.862
**Vomiting**	1 (7.7)	173 (48.1)	0.09 (0.01, 0.70)	0.01
**Diarrhoea**	3 (23.1)	66 (18.3)	1.34 (0.36, 4.99)	0.887
**Malaise**	1 (7.7)	30 (8.3)	0.92 (0.12, 7.29)	>0.999
**Cough**	3 (23.1)	110 (30.6)	0.66 (0.18, 2.53)	0.818
**Pre-hospital treatment with antibiotics or antimalarial**	5 (38.5)	89 (24.7)	0.9 (0.61, 5.97)	0.415
**Difficulty with breathing**	4 (30.8)	61 (16.9)	2.18 (0.65, 7.30)	0.349
**Oedema**	0	8 (2.2)	NA	>0.999
**Pallor of mucous membranes**	5 (38.5)	125 (34.7)	1.18 (0.38, 3.67)	0.991
**Jaundice**	0	34 (9.40)	NA	0.565
**Signs of dehydration**	1 (7.7)	39 (10.8)	0.67 (0.09, 5.43)	>0.999
**Bleeding manifestations**	4 (30.8)	4 (1.1)	39.56 (8.52, 187.7)	<0.001
**Lymphadenopathy**	0	12 (3.3)	NA	>0.999
**Unarousable coma**	0	17 (4.7)	NA	>0.999
**Hepatomegaly**	4 (30.8)	124 (34.4)	0.85 (0.26, 2.80)	>0.999
**Splenomegaly**	2 (15.4)	77 (21.4)	0.67 (0.15, 3.08)	0.913

OR (95% CI) = Odds Ratio (95% Confidence Interval) of feature in children with LVD versus other illnesses; LVD = Lassa virus disease; NA = not applicable.

### Outcomes

Twelve (3.2%) children with fever (3, 2.8% Cases and 9, 3.4% Controls; *p* >0.5) were discharged against medical advice. None had LVD. Thirteen (3.6%) of the 361 children whose hospital outcome was known died. Case fatality was 7/105 (6.7%) among the Cases and 6/256 (2.3%) among the Controls. The difference in case fatality rate did not attain statistical significance (*p* = 0.098). The deaths among the Cases included 1 child with LVD and 6 with malaria parasitaemia while the deaths among the Controls included 2 children with LVD, and 1 child each with malaria parasitaemia, meningitis, diabetic ketoacidosis, and myocarditis. Overall case fatality rate was 3/13 in children with LVD and 10/348 (2.9%) in children with other febrile illnesses. The difference was statistically significant (*p* = 0.017). Case fatality was 1/3 among the Cases versus 2/10 among the Controls with LVD (*p* >0.999).

## Discussion

Although seizures and coma have been described as acute neurologic morbidities of prognostic significance in LVD, few if any studies have systematically investigated the role of LVD in childhood CAWF, including its role in FC. Also, few if any studies in Nigeria have systematically investigated the role of LVD in acute febrile illnesses in childhood. This study found an overall LVD prevalence of 3.5% in febrile admissions to the CHER, 2.8% in CAWF, 3.8% in children with fever only, and 1.6% in FC versus 4.3% in non-FC. The corresponding rates in children with acute undifferentiated fever were 5.4% overall, 3.9% in children with CAWF, 6.0% in children with fever only, and 2.0% in children with FC versus 7.1% in those with NFC, respectively. These results show that while LVD is an important cause of fever and CAWF in children in endemic areas, there may be no relationship between its prevalence and clinical presentation in terms of presentation with fever alone versus fever with convulsions, and in terms of the nature of the CAWF, febrile versus non-febrile. This means that LVD may not be associated with convulsions in febrile children. One reason for this could be the relatively early presentation of many of the children with LVD—within 1 week of the onset of fever—whereas seizures and coma are late features in LVD [28,29]. It could also be that LVD, as with Ebola [49,50], is less severe in children. The reports of acute encephalopathy in LVD have been mainly in adults [28,29].

There may be a contrast between viral haemorrhagic fever viruses and other viruses as aetiologic agents of CAWF in childhood. Other viruses are commonly associated with FC in temperate countries [4], with the incidence rates of 5 common viruses in children with FC ranging from about 4–21% [5]. Convulsions are also common in both viral encephalitis [51,52] and bacterial meningitis [53]. Viral infections are also important causes of fever and FC in children in tropical countries [14,15,54]. In contrast, convulsions would appear to be uncommon manifestations of viral haemorrhagic fevers including Ebola and LVD, except perhaps in dengue infections where rates of up to 29.6% and 35% have been reported and up to 29.6% of patients “had one or more encephalitic signs, the most common being coma and convulsions” [55]. Mupere *et al* [56] reported convulsions in only 2.4% of Ugandan children and adolescents with Ebola and Erickson *et al* [49] noted that convulsions are late features of Ebola. Singh *et al* [57] reported convulsions in 8.9% of patients with Dengue fever. Sharp [21] reported a case series of 5 children with LVD, 4 of whom had convulsions that were described as febrile in 1. Four (15.4%) of 26 children with confirmed LVD in the prospective study of Webb *et al* [22] had convulsions. The rate of 3/13 (23.1%) in this study compares with the rate reported by Webb *et al* [22] (χ2 for the difference = 0.35). However, Webb *et al* [22] did not describe the nature of the convulsions in their patients.

The prevalence rates of LVD in the various groups of febrile children found in this study are less than the 7.6% prevalence of enteroviruses reported in Ibadan children with FC over 4 decades ago [14]. The rates are also less than the LVD prevalence of about 6% in febrile adults in endemic areas of Nigeria [26], and certainly less than the prevalence of 10–16% of adult medical admissions reported from endemic areas of Sierra Leone almost 3 decades ago [16]. It is however, comparable to the prevalence of 4.9% in febrile patients reported recently from a referral hospital in another endemic area of Sierra Leone [58]. The low prevalence rates found in this study should not, however, diminish from the importance of LVD in febrile children; unlike the situation with enteroviruses, effective treatment for LVD is available [33], LVD is associated with significant morbidity and mortality [48,59], and carries a high risk of nosocomial spread to other patients and health care workers [60,61]. LVD responds well to the broad-spectrum antiviral agent ribavirin [33], while it has been suggested that ribavirin could be useful in the treatment of the South American arenaviral infections [62] and Crimean Congo haemorrhagic fever [63,64]. However, the latter has not been confirmed and is controversial [65].

Familusi and Sinnette [14] reported a 7.6% prevalence of enteroviruses in Nigerian children with FC while Lewis *et al* [4] reported a 27% prevalence of several viruses in British children. Both of these studies involved the isolation of viruses from the CSF, aside from other sites in Lewis *et al*’s study [4]. The diagnosis of LVD was limited to RT-PCR testing of the serum in our study, but it is known that LASV could be present in the CSF while being absent in the serum of patients with acute central nervous system dysfunction [66,67]. In addition, the use of a combination of diagnostic testing increased the yield of cases in the report of Webb *et al* [22]. Furthermore, the use of newer molecular methods of diagnosis is associated with greater sensitivity [68]. Thus the additional testing of 300 RT-PCR-LASV negative samples bio banked in our centre between January 2012 and June 2013 using real time PCR resulted in the diagnosis of an additional 12 cases of LVD, giving a false negative rate of 4% on standard RT-PCR testing [Ogbaini-Emovon *et al*, personal communication 2015]. Thus, methodological differences could partly account for the relatively low prevalence of LVD in this study, and a combination of diagnostic methods and testing of multiple body fluids could have increased the yield of positive results. However, this limitation does not invalidate the results of the study. Rather, it could mean that the actual prevalence rates may be closer to the upper end of the 95% confidence intervals of the proportions found or even higher. Further studies are required to confirm this.

The prevalence of malaria parasitaemia was 46.3% among the Cases in this study. It was thus the commonest aetiologic agent of fever in children with CAWF but not in febrile children without convulsions in whom the prevalence was 14.0%. However, these rates confirm the leading role of malaria as a cause of fever and CAWF in endemic areas in the tropics [3,6–8,11,69]. Convulsions in malaria are both febrile and non-febrile in nature [70]. This can explain the higher prevalence of MP in children with CAWF, the 46.3% MP rate in whom compares with the 45.2% reported over 4 decades ago from Ibadan [6], but is higher than the rate reported from Benin City (32.5%) in midwestern Nigeria [71], and lower than the rates reported from Ilorin (71.7%) [69] and Jos (74.8%) [72], both of which are in the middle belt of Nigeria. The seeming lack of decline of the role of malaria should be of concern and could mean either that the control measures have been ineffective or that there is a resurgence of malaria. Seasonal transmission in the middle belt versus the all year round transmission in southern Nigeria can account for the differences in prevalence within Nigeria.

Four (31%) of 13 children with LVD in this study also had malaria parasitaemia and the prevalence of CAWF in children with LVD/malaria co-infection was significantly higher than that in those with LVD only. The implications of LVD/MP co-infection could be similar to that of the co-infection of malaria with serious bacterial infections in endemic areas. Among them is the risk of delayed or missed diagnosis of non-malaria infections [12,73,74]. Delayed or missed diagnosis of LVD could increase the risk of nosocomial transmission [60]. There is also the possibility that malaria parasitaemia could increase the risk of seeding of the brain by the virus and thus increase the risk of encephalitis and aseptic meningitis. Furthermore, the pathogenesis of seizures and coma in malaria could be additive to the pathogenesis of cerebral dysfunction in LVD. Further investigations are required to confirm the contribution of malaria to seizures and other disorders of cerebral function in LVD as suggested by the preliminary findings in this study.

The clinical differentiation of LVD from other common causes of fever is difficult [16,18,27]. The findings in this study are not different, and the common symptoms of fever, abdominal pain, sore throat, cough and gastroenteritis are not helpful in diagnosis. Also, even though bleeding manifestations and lack of vomiting were significantly associated with increased prevalence of LVD among our patients, the sensitivities of the 2 features (lack of vomiting 7.7% and bleeding 30.8%) are very low to be of practical importance in diagnosis. These findings further emphasize the utmost need for a high index of suspicion in diagnosis.

The case fatality rates (CFR) in LVD and CAWF in this study are worth discussing. About 23% of the children with LVD and 3% with other illnesses died. A CFR of 23% in the era of availability of treatment with ribavirin is quite high and unacceptable but it is congruent with the evidence that LVD is a highly fatal illness [19,48,59,75]. The CFR in children with CAWF in this study was about 7% versus a CFR of only about 2% in febrile children without convulsions. One of the 7 deaths had LVD while 6 had cerebral malaria. The CFRs in CAWF reported from other studies in tropical Africa over time range from as low as 1.8%-2.5% in Ilorin [69] and Jos [72], Nigeria through 6.4% in Ibadan, Nigeria [6] to as high as 27% in Uganda [7]. The latter might have been related to the high frequency of cerebral malaria, septicaemia and acute and chronic CNS infections in the Ugandan series.

Two other findings in this study are also worth discussing: the prevalence of CAWF among febrile admissions to the CHER, the prevalence of CAWF among the admissions to the CHER, and the prevalence of undiagnosed fever in children with acute fevers. The 11.8% prevalence of CAWF among admissions to the CHER, which was 29% of the febrile admissions, is near the intermediate point of the 3–28% prevalence range reported from earlier studies [2,6,71]. Our findings nonetheless emphasize the contribution of CAWF to childhood emergencies including neurologic emergencies in the tropics.

The cause(s) of fever was undiagnosed by a combination of clinical examination and laboratory methods in 39.4% of febrile children in this study, in 25.0% of the Cases and 45.3% of the Controls. These rates are within the range reported from previous studies of children with CAWF and other febrile illnesses [6–8,12]. The limitations in investigative resources, which have unfortunately been longstanding, are a major factor in the far higher prevalence of undiagnosed fevers in developing countries in the tropics [12,76,77], although it should also be noted that even in resource-abundant settings, it is not always possible to diagnose the cause of fever. However, we acknowledge that more comprehensive investigation of the cause/s of fever among our patients using contemporary methods [68,78] could have considerably reduced the number of cases of undiagnosed fevers and also enabled more specific diagnosis beyond the reliance on clinical methods. Further studies are planned to address this. Still, although undiagnosed fevers are often due to common viral and bacterial infections [12,76], the results of this and other studies demonstrate that other infectious diseases should also be considered.

Perhaps related to the currently poor state of diagnostic resources in many developing countries in Africa is the inability to confirm the diagnosis of bacterial meningitis in CSF samples with typical changes in this study, hence the term possible bacterial meningitis [43] was adopted. The success rate in determination of the causative organisms in children with typical CSF changes in a previous study in our centre was 44.1% [79] but none of the children in the present study had a positive blood culture, CSF Gram stain or CSF culture. The abysmally poor rate in this study is difficult to explain. Although about 25% of the patients admitted to a history of pre-hospital treatment with antibiotics, and this is likely to be an underestimate bearing in mind previous experiences in the same practice area [80], and partial treatment with antibiotics can markedly reduce the success rates of routine bacteriologic methods of diagnosis [81,82], the poor results of both Gram stain and culture in this study is unusual. Other workers [82] have drawn attention to the challenges in the management of bacterial meningitis in resource-poor settings, among which are those of pre-hospital treatment with antibiotics and limitations in diagnostic resources, and these issues may have worsened. Still, regarding the findings from this study, it should be worthwhile to remark that the role of bacterial meningitis and bacteraemia in febrile admissions and CAWF was not the focus of the study. Rather, the focus was on the role of LVD, and a follow up of the role of malaria parasitaemia after several decades. Therefore, the poor results of bacteriologic tests in the study should not diminish from the importance of the findings.

Finally, it is perhaps also important to discuss adoption of the descriptive term LVD as well as the need for the classification of cases of CAWF into febrile and non-febrile convulsions. We adopted for use in this report the more inclusive description LVD rather than the more usual but somewhat restrictive terms Lassa haemorrhagic fever and Lassa fever, in recognition of the varied nature of the manifestations of infection with the LASV, which spectrum ranges from sub-clinical or mildly symptomatic or even asymptomatic infections to severe infections, sometimes with haemorrhagic manifestations [16,59,83]. This is not unlike the evolution of the terminology for the description of infection with the Ebola virus from the earlier descriptions as Ebola fever and Ebola haemorrhagic fever to the current one of Ebola virus disease for the same reasons. Bleeding in both Ebola and LVD are a marker of disease severity [48,59,84].

The classification of childhood CAWF into febrile and non-febrile convulsions is of practical importance, with implications for both the approach to management and outcome [1,38]. It also has a developmental basis. However, recourse to the classification was mostly de-emphasized in this paper in order not to distract from the narrative of the overall pattern of illnesses in the Cases versus Controls.

### Conclusion

LVD is an important cause of fever, including undifferentiated fever in children in endemic areas, but it is not significantly associated with convulsions in febrile children. Its prevalence, and lack of clinical differentiation on presentation, underscores the importance of a high index of suspicion in diagnosis. Screening of febrile children with undifferentiated fever in endemic areas for LVD could be an important medical and public health control measure.

## Supporting information

S1 Text Clinical data form. (ZIP)
