# Supplementary figures and images for: Prevalence of Lassa Virus Disease (LVD) in Nigerian children with fever or fever and convulsions in an endemic area
Source: PLoS Negl Trop Dis. 2017 Jul 3;11(7):e0005711. doi: 10.1371/journal.pntd.0005711 (PMC5510890; doi:10.1371/journal.pntd.0005711)

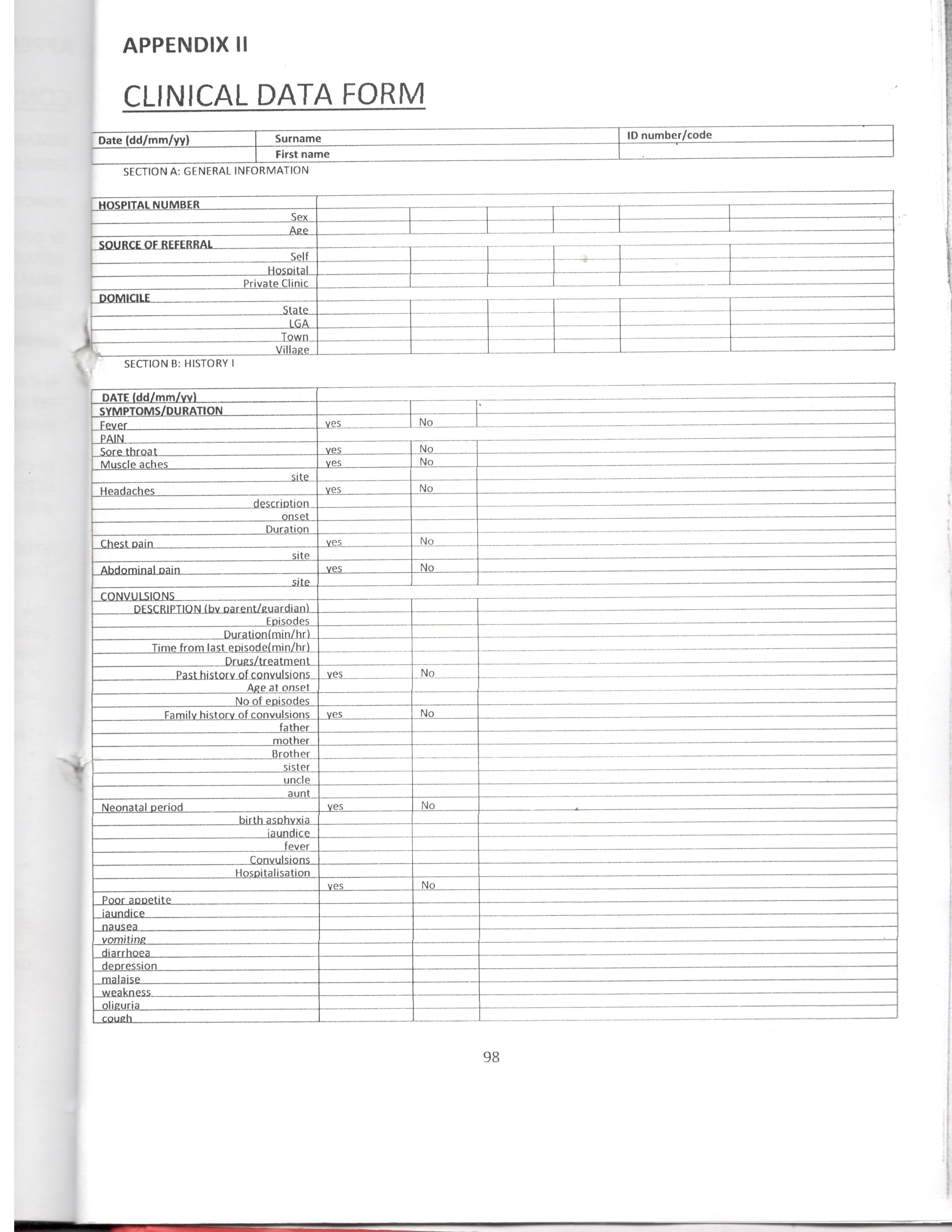

Supplement: S1 Text — (ZIP) [file pntd.0005711.s001.zip › S1 Text. Clinical data form/S1 Text. Clinical data form.jpeg]

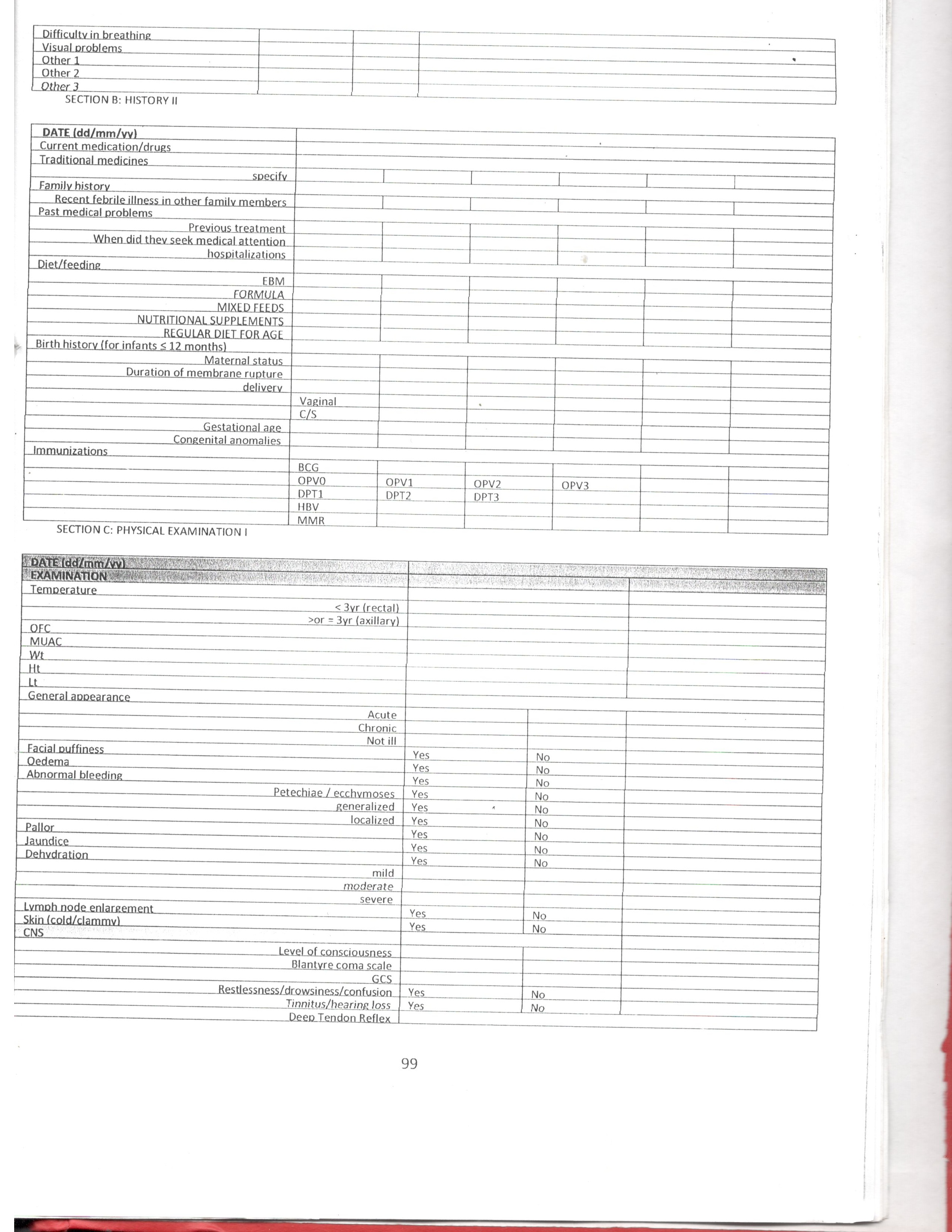

Supplement: S1 Text — (ZIP) [file pntd.0005711.s001.zip › S1 Text. Clinical data form/S2 Text. Clinical data form.jpeg]

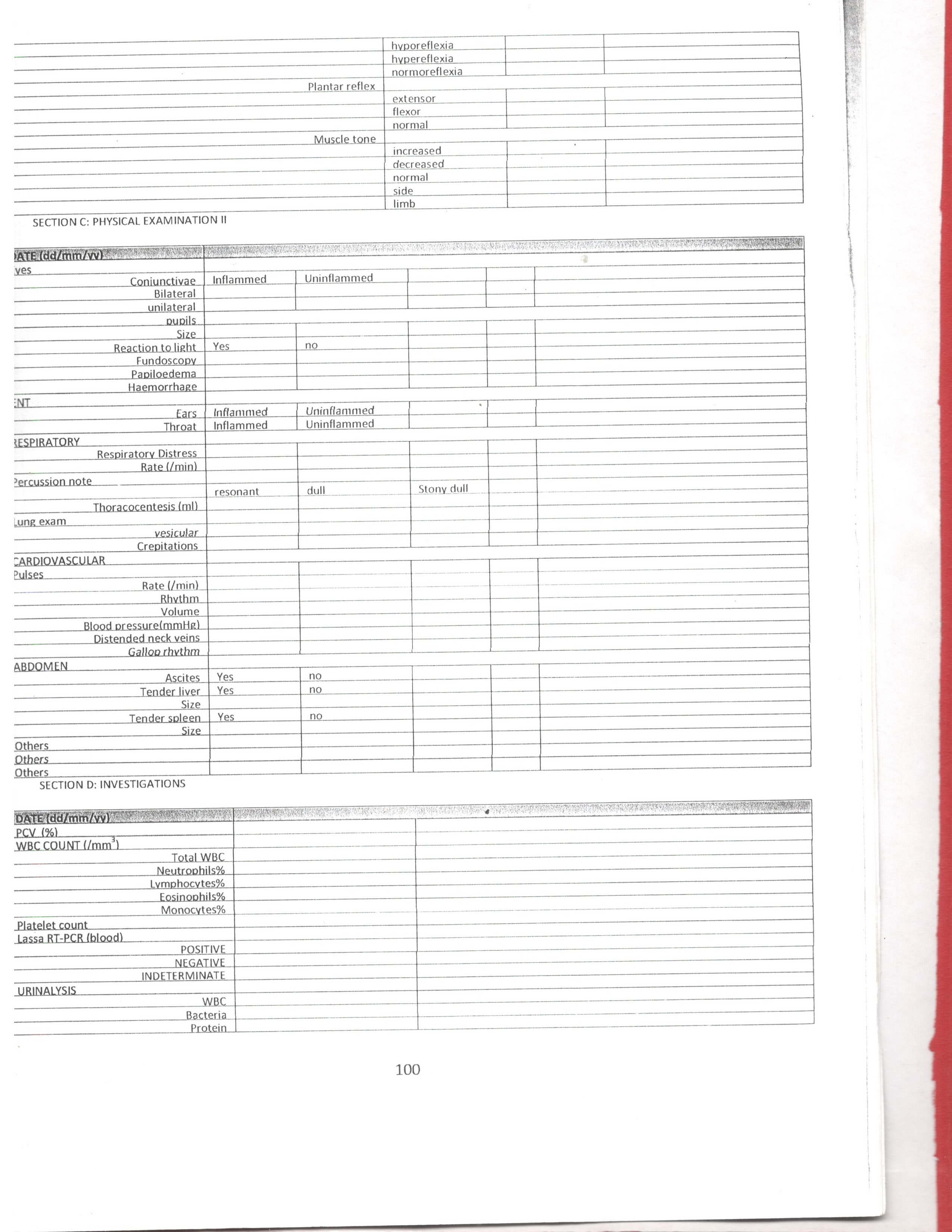

Supplement: S1 Text — (ZIP) [file pntd.0005711.s001.zip › S1 Text. Clinical data form/S3 Text. Clinical data form.jpeg]

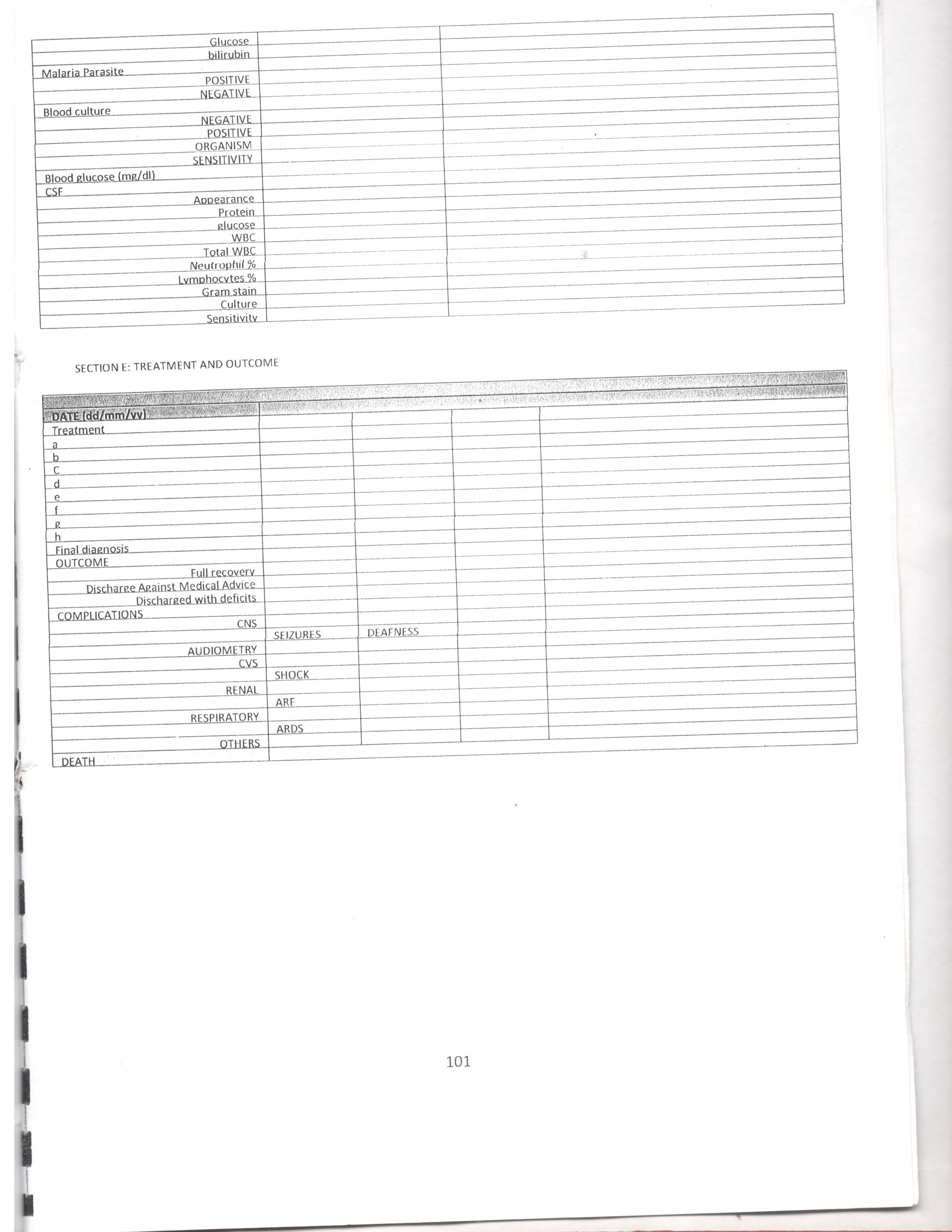

Supplement: S1 Text — (ZIP) [file pntd.0005711.s001.zip › S1 Text. Clinical data form/S4 Text. Clinical data form.jpeg]
